# Yishen capsule promotes podocyte autophagy through regulating SIRT1/NF-κB signaling pathway to improve diabetic nephropathy

**DOI:** 10.1080/0886022X.2020.1869043

**Published:** 2021-01-11

**Authors:** Yuxiang Liu, Wenyuan Liu, Ziyuan Zhang, Yaling Hu, Xiaodong Zhang, Yanyan Sun, Qingqing Lei, Dalin Sun, Ting Liu, Yanjun Fan, Hui Li, Wujie Ding, Jingai Fang

**Affiliations:** a The First College for Clinical Medicine, Shanxi Medical University, Taiyuan, People’s Republic of China; b Department of Nephrology, The First Hospital of Shanxi Medical University, Taiyuan, People’s Republic of China

**Keywords:** Diabetic nephropathy, SIRT1/NF-kB pathway, podocyte, autophagy, Yishen capsule

## Abstract

Diabetic nephropathy (DN) is a common complication of diabetes. Yishen capsule, composed of Chinese herbs, improves the clinical outcome in DN patients. However, its therapeutic potential and underlying mechanisms require further elucidation. Hence, our study aimed to investigate the underlying mechanisms and therapeutic potential of Yishen capsule in DN. Streptozotocin-induced DN rats were treated with Yishen capsules (1.25 g/kg/day) for 8 weeks. Then, blood glucose and urine protein levels were measured. Hematoxylin and eosin staining and western blot assays were used to examine the histologic changes and gene expression, respectively, in kidney samples. Mouse podocytes were treated with rat serum containing Yishen capsule and transmission electron microscopy was used to examine autophagosome formation. Cell counting kit-8 assay was performed to examine cell proliferation. Western blot and reverse transcription-quantitative polymerase chain reaction (RT-qPCR) analyses were conducted to detect changes in gene expression. The localization of SIRT1 was examined in the podocytes using immunocytofluorescence assay. We found that Yishen capsule relieved pathological changes, decreased urine protein, increased SIRT1, LC3-II, and Beclin-1 expression, and reduced acetylated NF-κB p65 expression *in vivo*. In addition, rat serum containing Yishen capsule showed improved podocyte proliferation, promoted the mRNA and protein levels of LC3-II and Beclin-1, and induced nuclear translocation of SIRT1. Furthermore, it increased SIRT1 expression and decreased mRNA level of NF-κB in the serum. SIRT1 inhibitor increased the mRNA level of NF-κB. Our data suggests that Yishen capsule improves DN by promoting podocyte autophagy *via* the SIRT1/NF-κB pathway.

## Introduction

Diabetic nephropathy (DN) is the main cause of end-stage chronic kidney disease, which seriously affects the quality of life of patients. Podocyte injury is a major contributor to the development of DN and proteinuria [1]. Recent studies have shown [2,3] that autophagy is an important mechanism that regulates cell growth, maturation, differentiation, and death, and plays a significant role in the structural and functional stability for podocytes. Regulation of the autophagic process may be a key target in the treatment of DN.

Yishen capsule is a traditional Chinese medicine developed based on several decades of clinical practice at the First Affiliated Hospital of the Shanxi Medical University. The main components of Yishen capsule are *Astragalus membranaceus*, *Angelica sinensis*, *Euryale ferox*, *Alisma orientalis*, and *Rhodiola rosea*, as shown in Table 1. Modern pharmacological studies have found that *A. membranaceus* not only inhibits inflammation and regulates body immunity [4], but also ameliorates high glucose-induced podocyte apoptosis [5]. *E. ferox* inhibits inflammation and delays DN progression [6]. *A. orientalis* has vasodilating, immunomodulatory, and diuretic functions [7]. *A. sinensis and R. rosea* have antioxidative stress properties and improve microcirculation [8]. Clinically, Yishen capsule significantly reduced the level of urine protein in DN patients [9], and improved the clinical outcome of patients. Moreover, in a rat model of DN induced by unilateral nephrectomy and intraperitoneal injection of streptozotocin (STZ), Yishen capsule was found to restore podocyte foot process effacement and expression of podocalyxin [10]. Furthermore, Yishen capsule enhanced the expression of nephrin and podocin in podocytes under high glucose conditions *in vitro* [11]. However, the mechanisms by which Yishen capsule regulates autophagy and DN remains unclear.

**Table 1.** Components of the Yishen capsule and their scientific (Latin binomial nomenclature) names.

Components of Yishen capsule	Full scientific species name
*A. membranaceus*	*Astragalus propinquus* Schischkin
*A. sinensis*	*Radix Angelica sinensis (Oliv.) Diels*
*E. ferox*	*Nymphaeaceae Euryale ferox* Salisb.
*A. orientale*	Alisma orientale (Sam.) Juzep.
*R. rosea*	*Rhodiola crenulata* (HK. f. et.Thoms) H. Ohba

Sirtuin type-1 (SIRT1) is a member of the silent information regulator 2 (Sir2)-like family of proteins [12]. It is a highly conserved protein during evolution. SIRT1 regulates the activities of many proteases by regulating their deacetylation. Specific knockdown of SIRT1 in podocytes induced severe podocyte damage and increased proteinuria, whereas kidney damage was significantly alleviated after SIRT1 overexpression in diabetic mice [13]. Therefore, the expression of SIRT1 in podocytes influences the occurrence and development of DN. SIRT1 reportedly deacetylates nuclear factor-kappaB (NF-κB) p65, which inhibits the transcription of downstream factors and suppresses inflammatory response [14]. Moreover, NF-κB p65 upregulates the expression of autophagy-related proteins, such as LC3II and Beclin-1 [15]. Therefore, the current study aimed to investigate whether Yishen capsule improves DN by regulating the SIRT1/NF-κB pathway to enhance podocyte autophagy.

## Materials and methods

### Animals and cells

Seventy male Sprague-Dawley (SD) rats weighing 185–215 g were purchased from the Experimental Animal Center of the Shanxi Medical University and housed in the animal room of the Shanxi Medical University. The animal certificate number was SCXK (Shanxi) 2015-0001. Immortalized mouse podocytes (MPC5) were kindly provided by Dr. Guizhi Sun at Monash University, Australia and were cultured in RPMI 1640 medium supplemented with 10% fetal bovine serum and 50 U/L penicillin–streptomycin, under a humidified atmosphere with 5% CO_2_ at 37 °C.

### Chemicals and reagents

Yishen capsules were purchased from the Department of Pharmacy of the First Hospital of the Shanxi Medical University. It was composed of *A. membranaceus*, *A. sinensis*, *E. ferox, A. orientale, and R. rosea* in a ratio of 3:2:3:2:1. The preparation involved boiling the herbs in water thrice for 1 h. The decoctions were combined and filtered through a 3 M membrane. The decoction was then concentrated through vacuum evaporation to a density of 1.20–1.24 at 70 °C. The concentrated decoction was further spray-dried into particles that were subsequently filled into capsules.

Rabbit anti-SIRT1, anti-NF-κB p65, and anti-Beclin-1 antibodies were purchased from Cell Signaling Technology (Denver, MA). LC3 antibody was purchased from Sigma (St. Louis, MO). Rabbit anti-Nephrin, rabbit anti-NF-kB p65 K310 acetylation, and β-actin antibodies were purchased from Abcam (USA). Horseradish peroxidase-labeled secondary antibody, FITC-labeled secondary antibody, and CCK8 kit were obtained from Wuhan Boster Biological Technology Co. Ltd (Wuhan, China). Fetal bovine serum was purchased from Gibco (USA). Dulbecco’s modified Eagle’s medium was purchased from Hyclon (USA). Reverse transcription and qPCR kits were purchased from Promega (USA). Resveratrol and Ex-527 were purchased from MCE (USA). Sodium carboxymethylcellulose (CMC), STZ, and sodium citrate buffer were purchased from Solarbio (China). Urine protein detection kit was purchased from Jianglai Bio (Shanghai, China).

### Establishment of DN rat model and Yishen capsule intervention

Fifty SD rats were acclimatized in the animal room of the Shanxi Medical University for 1 week prior to the experiment. The rats were then randomly divided into two groups: the control (*n* = 10) and model groups (*n* = 40). The rats in the model group underwent surgery to remove their left kidney after anesthesia with 10% chloral hydrate, while the rats in the control group underwent sham-surgery to remove the kidney capsule. One week after the surgery, the rats in the model group were intraperitoneally injected with STZ (50 mg/kg), while those in the control group were injected with the same amount of sodium citrate buffer. Rats with a blood glucose level of 16.7 mmol/L or higher, and 24-h urine volume of 50 mL or higher were considered as being successfully established DN rat models. These DN model rats were further randomly assigned into three groups: diabetic nephropathy group (group DN, *n* = 10, treated with normal saline by gavage for 8 weeks), Yishen capsule group (group YS, *n* = 10, Yishen capsule treatment by daily gavage at a dose of 1.25 g/kg for 8 weeks), and resveratrol group (group R, *n* = 10; resveratrol treatment by daily gavage at a dose of 100 mg/kg for 8 weeks). The urine volume, water consumption, random blood glucose level, and urine protein amount were measured and recorded at the beginning and the end of the gavage period.

### Histological examination

At the end of the gavage period, the rats were sacrificed and kidney samples were harvested. The samples were fixed in 4% paraformaldehyde (PFA), embedded in paraffin, and cut into 5 μm thick sections. After drying, the sections were stained with hematoxylin and eosin (H&E) and examined under a microscope. Five non-overlapping fields of view under 100× and 200× optical microscopes were randomly selected and analyzed. Double-blind analysis and scoring were performed by two nephrologists. The scoring criteria for glomerular injury were as follows: Thickening of glomerular basement membrane with nonspecific or mild variations was scored as 0; mild mesangial expansion was scored as 1; severe mesangial expansion without tuberous sclerosis was scored as 2; appearance of tuberous sclerosis was scored as 3; observation of glomerulosclerosis in more than 50% of glomeruli was scored as 4. The scoring criteria for renal interstitial fibrosis and tubular atrophy (IFTA) were as follows: 0, absent; 1, <25%; 2, 25–50%; 3, >50%; and 4, >75% of the total area. The scoring criteria for interstitial inflammation were as follows: 0, absent; 1, inflammation only in relation to IFTA; and 2, inflammation in areas without IFTA. Arteriolar hyalinosis was scored as follows: 0, absent; 1, arteriolar hyalinosis in one site; and 2, arteriolar hyalinosis in more than two sites. Arteriosclerosis was scored as follows: 0, no intimal thickening; 1, intimal thickening less than thickness of media; and 2, intimal thickening greater than thickness of media.

### Preparation of Yishen capsule serum

Twenty male SD rats were randomly divided into the following groups (*n* = 5 each): control group received normal saline (N), low Yishen capsule group received 0.625 g/kg/day of Yishen capsule (LYS), medium Yishen capsule group received 1.25 g/kg/day of Yishen capsule (MYS), and high Yishen capsule group received 2.5 g/kg/day of Yishen capsule (HYS). Rats received the treatment through gavage once a day for 7 days. Two hours after the last gavage, the rats were anesthetized by intraperitoneal injection with 10% chloral hydrate (350 mg/kg body weight). Blood was collected through the abdominal aorta, and serum was isolated by centrifuging for 10 min at 2000 rpm at room temperature. The complement was inactivated by incubation in a 56 °C water bath for 30 min. After sterilization by passing through a sterilized filter membrane (0.22 μm), serum was aliquoted and stored at −20 °C until use. Animals were euthanized by intraperitoneal injection of pentobarbital after anesthesia. The animal experiments in this study were approved by the Animal Ethics Committee of the Shanxi Medical University (2019LL242). All procedures were performed in accordance with the Guidelines for the Care and Use of Laboratory Animals.

### Experimental design using mouse podocytes

Experimental groups were set as follows: normal glucose (NG, 5.5 mmol/L glucose + 10% normal rat serum); high glucose (HG, 30 mmol/L glucose + 10% normal rat serum); Yishen capsule serum group (HG + YS, 30 mmol/L glucose + 10% Yishen capsule serum), high glucose + resveratrol group (HG + R, 30 mmol/L glucose + 10 mmol/L resveratrol + 10% normal rat serum), high glucose + SIRT1 inhibitor group (HG + Ex, 30 mmol/L glucose + 2 μM Ex-527 + normal rat serum).

### Cell proliferation assay

Podocytes were plated in 96-well plates at 300 cells/well (*n* = 6) and cultured in an incubator overnight. The next day, the culture medium was discarded and fresh culture media (100 µL) containing different interventions was added to each well. After treatment for 24 h, CCK-8 reagent (10 μL/well) was added into each well. Cells were incubated with CCK-8 reagent for 1 h, and then the absorbance values were measured at a wavelength of 450 nm.

### Immunocytofluorescence assay

Podocytes (3000 cells/well) were plated on culture slides placed in a six-well plate. After the cells adhered to the slides, they were incubated with culture media containing different interventions for 24 h. After washing with phosphate-buffered saline (PBS), the cells were fixed with 4% PFA at room temperature for 30 min, followed by incubation with 0.5% Triton X-100 at room temperature for 5 min. After washing with PBS, cells were blocked with 3% BSA in PBS for 30 min and incubated with antibodies against the target proteins at 4 °C overnight. Cells were then washed with PBS and incubated with FITC-conjugated secondary antibodies at room temperature for 2 h, followed by DAPI staining for 5 min in the dark. After washing with PBS, the slides were sealed with 90% glycerin and observed under a confocal microscope.

### Western blotting (WB) assay

Cells or kidney tissues were lysed in lysis buffer and protein concentration was determined using the BCA assay. Proteins were separated by 10% sodium dodecyl sulfate polyacrylamide gel electrophoresis (SDS-PAGE) and then transferred to the polyvinylidene difluoride (PVDF) membrane using a Bio-Rad western blot transfer system. After blocking with 5% nonfat dry milk in TBS for 1 h at room temperature, the samples were incubated with the following rabbit antibodies: anti-SIRT1, anti-Beclin-1, anti-LC3, anti-Nephrin, anti-NF-kB p65 K310, anti-NF-κB p65, and anti-β-actin antibodies; all at 1:1000 dilution) at 4 °C overnight. After washing with TBS-T, the membranes were incubated in horseradish peroxidase-labeled secondary antibody (sheep anti-rabbit antibody, 1:500) at room temperature for 1 h. After washing with TBS-T, the protein bands were visualized using ECL chemiluminescence reagent.

### RT-qPCR analysis

Total RNA was extracted using the TRIzol method, and 1 µg of total RNA was reverse transcribed into cDNA using a reverse transcription kit. Target genes were then amplified using a 7500 real-time PCR system (Applied Biosystems, Life Technologies) according to the following cycling conditions: predenaturation at 95 °C for 10 min; 40 cycles of denaturation at 95 °C for 15 s and annealing at 60 °C for 1 min. The expression of target genes was normalized to that of β-actin. Relative mRNA expression was analyzed using the 2^−ΔΔCt^ method. Primers were designed on the PubMed website and synthesized by Invitrogen. Primer sequences for the target genes were as follows:

SIRT1-forward: 5′- CGGCTACCGAGGTCCATATAC -3′; 135bp
SITR1- reverse: 5′- ACAATCTGCCACAGCGTCAT -3′;
NF-kB p65-forward: 5′- CGCGCCGCGCATTTC-3′; 114bp
NF-kB p65- reverse: 5′- TGAGGGGAAACAGATCGTCC -3′;
LC3-forward: 5′- TTGGTCAAGATCATCCGGCG-3′; 163bp
LC3- reverse: 5′- TGGGAGGCGTAGACCATGTA-3′;
Nephrin-forward: 5′- AGCTACCCTGCATAGCCAGA-3′; 84bp
Nephrin- reverse: 5′- CCCAAGCTATGGACACTGGT-3′;
Beclin-1-forward: 5′-ATGGAGGGGTCTAAGGCGTC-3′; 125bp
Beclin-1- reverse: 5′- TGGGCTGTGGTAAGTAATGGA-3′;
β-actin-forward: 5′-ACCCCTTCCAAGAAGAGCAG-3′; 154bp
β-actin- reverse: 5′-CAGATCTTGAGCTCGGCAGT-3′.

### Transmission electron microscopic (TEM) examination

Podocytes were incubated with different interventions in culture media for 24 h, and then the cells were washed with pre-cooled PBS and digested. The cells were collected and fixed in a mixture of 2.0% PFA and 2.5% glutaraldehyde overnight at 4 °C. After fixing, the cells were incubated in 1% aqueous osmium tetroxide and after five washes in cacodylate buffer (5 min each), the samples were dehydrated through a series of graded alcohols and embedded in Epon 812 medium. Semi-thin sections were cut on a microtome (Ultracut Reichert-Jung, Leica Microsystems, EM-UC7, Germany) and stained with uranyl citrate for 15 min. After washing, the samples were incubated with lead citrate for 7 min. Ultra-thin sections were cut with a diamond knife (Diatome, 80-HIS, Switzerland) and transferred to copper grids. Samples were then examined under a transmission electron microscope (Nippon Electronics).

### Statistical analysis

All data were expressed as mean ± standard deviation. Comparisons between groups were performed using one-way ANOVA, and pairwise comparisons between groups were performed using the LSD-t test in SPSS 22.0 software. *p* < 0.05 was considered statistically significant. Graphs were drawn using GraphPad Prism (version 5.0 for Windows).

## Results

### Establishment of the DN rat model

A rat in the model group died of infection 2 days after nephrectomy and another one died of toxicity caused by the drug 3 days after STZ injection. Thirty-six rats with DN model were successfully established and 30 of them were further divided into three groups: DN, YS, and R groups. During the experiment, two rats in the DN group died after 4 and 7 weeks of feeding, respectively. One rat in the YS group died after 6 weeks of feeding, and one rat in the R group died after 4 weeks of feeding. In the model group, the random blood glucose levels of rats were higher than 16.67 mmol/L and the urine output was more than 50 mL/24 h, which was higher than that in the N group.

### Yishen capsule reduced the urine output and urine protein level in DN rats

**Figure 1.**
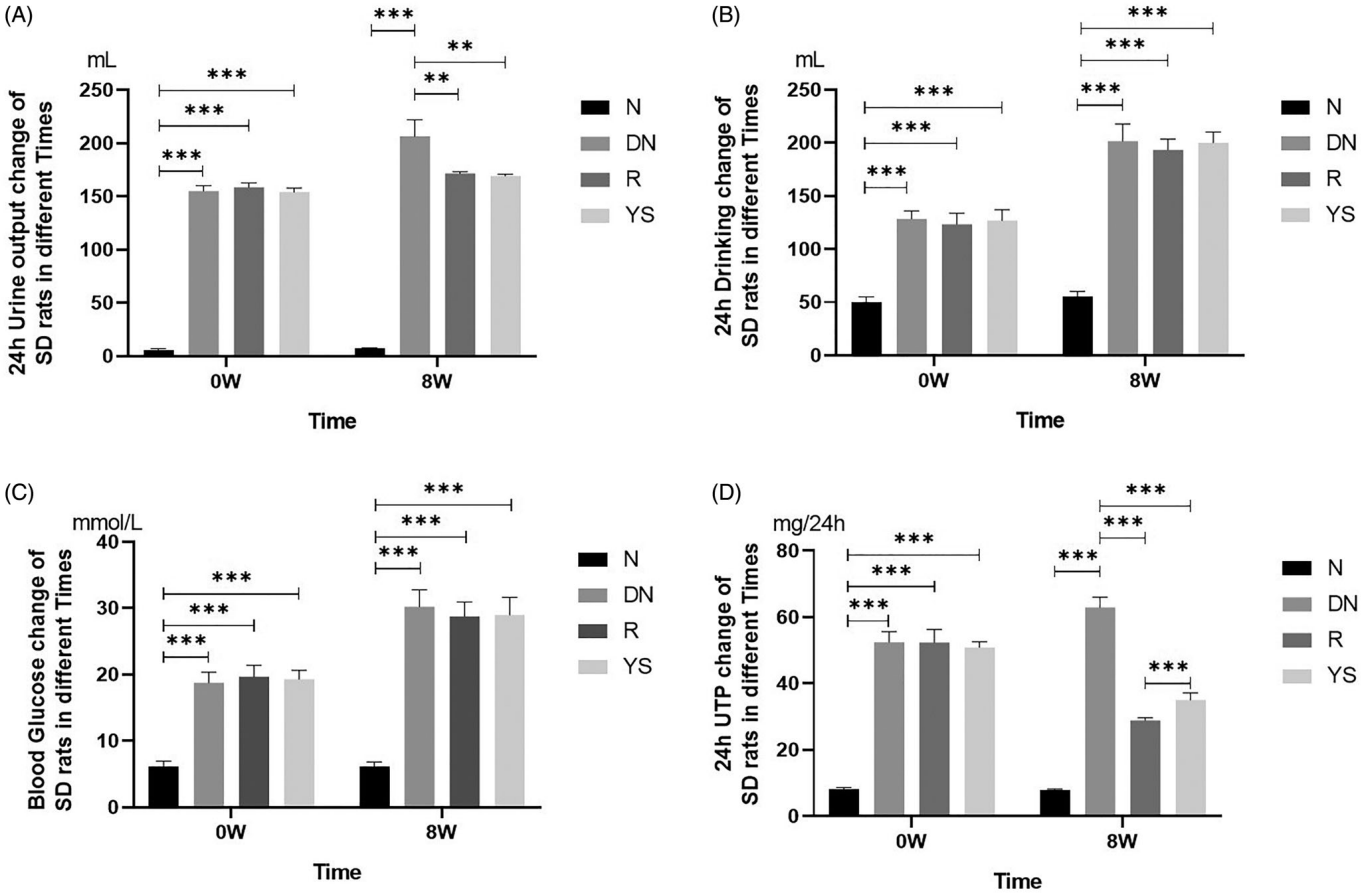
Effects of Yishen capsule on physiological index in rats with DN. (A) 24-h urine output; (B) Water intake; (C) Random blood glucose level; (D) 24 h urinary total protein (24 h UTP). Values are presented as the mean ± SD. *n* = 8–10. **p < 0.01; ***p < 0.001.

Prior to treatment with resveratrol and Yishen capsule (expressed as 0 weeks), there was no significant difference in terms of urine output, water intake, blood glucose level, and urine protein level among the DN, R, and YS groups (Figure 1(A–D), left). After 8 weeks, the condition of the rats in the DN group gradually worsened, including loss of weight and hair, decreased mobility and responsiveness, increased diet and water intake, and increased urine output. Both the Yishen capsule and resveratrol treatments improved the health condition of rats as evidenced by decreased urine output and urine protein level (Figure 1). Notably, the 24 h urinary total protein (24 h UTP) in the R group was significantly lower than that in the YS group (Figure 1(D)) after 8 weeks. However, neither resveratrol nor Yishen capsule decreased the level of blood glucose or water intake in rats (Figure 1).

### Yishen capsule relieved the histologic changes in DN rat kidneys

**Figure 2.**
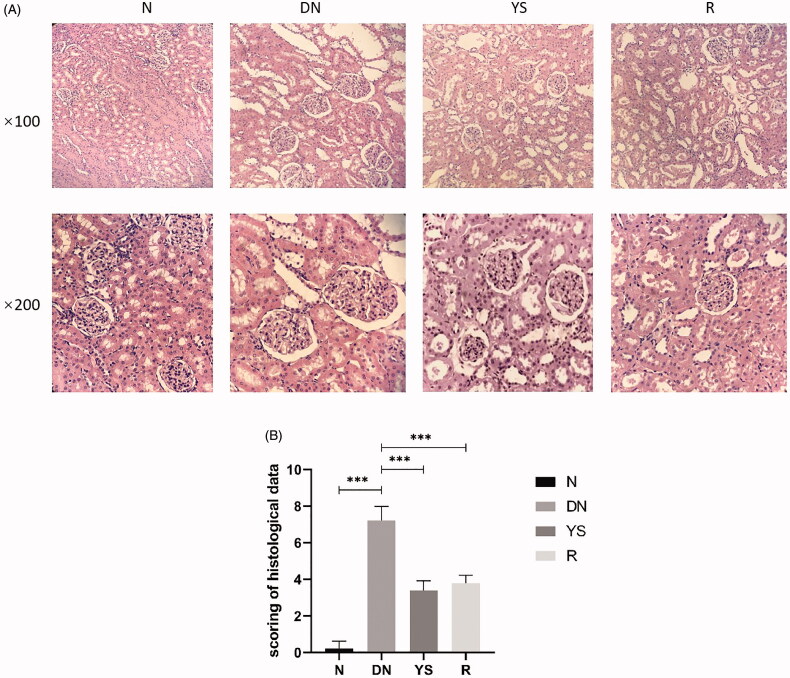
Effect of Yishen capsule on histological changes in the kidney of rats. (A) Representative histological images of sections from different groups at 100× and 200× magnification using an optical microscope. (B) Renal pathological scores. Values are presented as the mean ± SD. *n* = 10.

Glomerular morphology and structures of the mesangial and basement membranes were normal in the N group. The glomeruli were markedly enlarged and mild hyperplasia was observed in the mesangial and basement membranes in the DN group. In addition, vacuolar degeneration of the basement membrane and hypertrophy of renal tubular epithelial cells were observed in the DN group. Compared to the DN group, the YS group showed reduced glomerular volume and alleviation in mesangial hyperplasia and basement membrane thickening, similar to the R group (Figure 2(A)). Renal pathological scores confirmed the efficacy of Yishen capsule in relieving histologic changes (Table 2; Figure 2(B)).

**Table 2.** Renal pathological scores.

	N group (*n* = 10)	DN group (*n* = 10)	R group (*n* = 10)	YS group (*n* = 10)
Glomerular injury (0–4)	0 ± 0.0	1.3 ± 0.48	0 ± 0.00	0 ± 0.0
Interstitial fibrosis and tubular atrophy (IFTA) (0–4)	0.1 ± 0.32	2.2 ± 0.42	1.9 ± 0.32	1.5 ± 0.53
Interstitial inflammation (0–2)	0.1 ± 0.32	1.2 ± 0.42	0.3 ± 0.48	0.6 ± 0.52
Arteriolar hyalinosis (0–2)	0 ± 0.00	1.5 ± 0.53	0.9 ± 0.32	1.0 ± 0.00
Arteriosclerosis (0–2)	0 ± 0.00	1.0 ± 0.00	0.7 ± 0.48	0.3 ± 0.48

Values are presented as the mean ± SD. *n* = 10.

### Effect of Yishen capsule on the expression of SIRT1, acetylated NF-κBp65, LC3-II, and beclin-1 in DN rat kidneys

**Figure 3.**
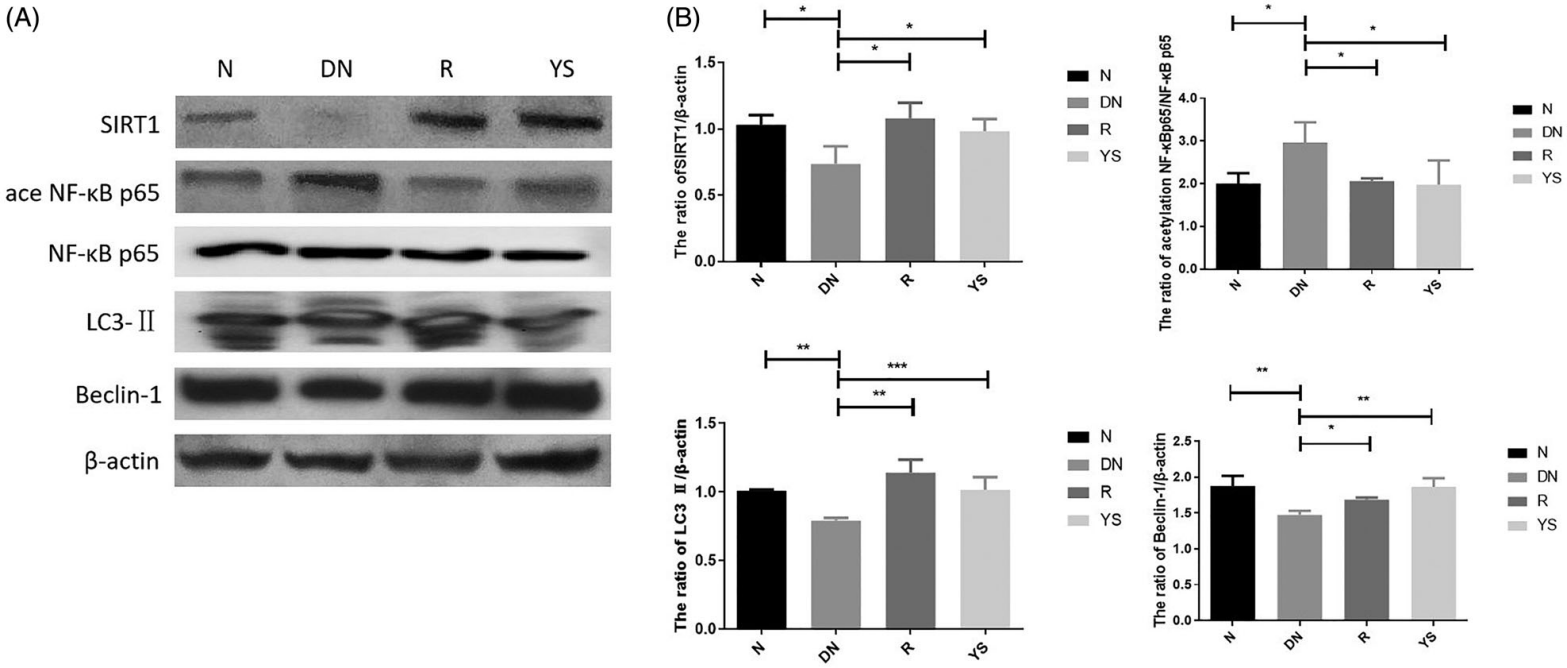
Effects of Yishen capsule on the expression of SIRT1, acetylated NF-κB p65, NF-κB p65, LC3-II, and Beclin-1 in the kidney of rats. (A) Representative western blotting images showing the expression of SIRT1, acetylated NF-κB p65, LC3-II, and Beclin-1; (B) Quantification of SIRT1, acetylated NF-κB p65, NF-κB p65, LC3-II, and Beclin-1 expression. Values are presented as the mean ± SD. *n* = 3. *p < 0.05; **p < 0.01; ***p < 0.001.

WB was performed to analyze the expression of SIRT1, Beclin-1, LC3-II, and acetylated NF-κBp65 in the kidney of rats. As shown in Figure 3, the expression levels of SIRT1, Beclin-1, and LC3-II were downregulated whereas that of acetylated NF-κBp65 was upregulated in the DN group. However, Yishen capsule and resveratrol increased the expression of SIRT1, Beclin-1, and LC3-II expression, and decreased NF-κBp65 expression (Figure 3).

### Expression of SIRT1 and LC3-II in mouse podocytes

**Figure 4.**
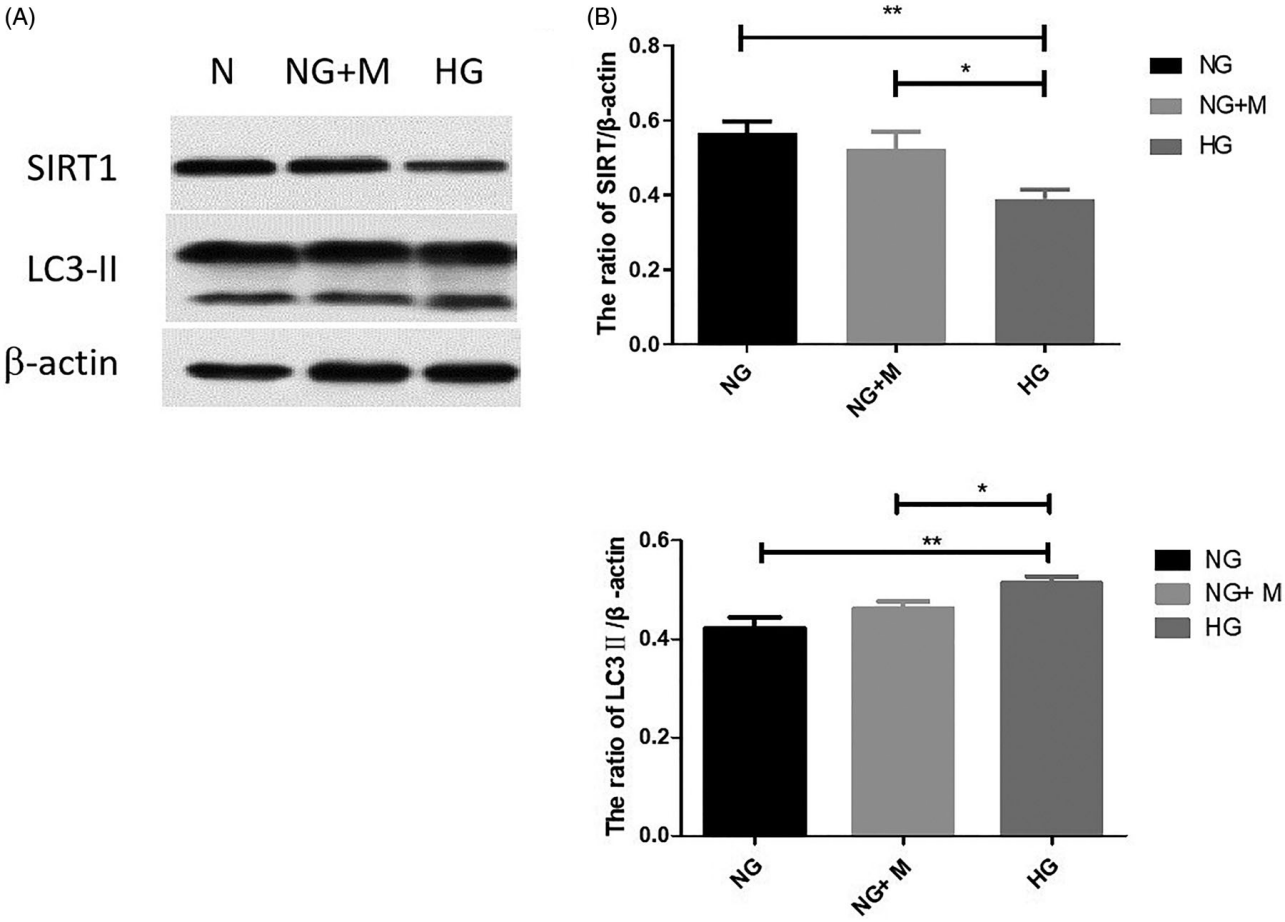
Expression of SIRT1 and LC3-II in immortalized mouse podocytes under normal glucose (NG), high glucose (HG), and mannitol isotonic (NG + M) conditions. (A) Representative western blotting bands corresponding to SIRT1, LC3-II, and β-actin. The expression trend of LC3II is indicated by the lower one of two bands in Western Blot, the upper band demonstrates the expression of LC3I. (B) Quantification of SIRT1 expression; (C) Quantification of LC3-II expression. Values are presented as the mean ± SD. *n* = 3. *p < 0.05; **p < 0.01.

To investigate the mechanisms underlying autophagy in podocytes, the expression of SIRT1 and LC3-II was examined. As shown in Figure 4, SIRT1 expression was decreased whereas that of LC3-II was increased with an increase in glucose level in the culture medium. There was no significant difference between the mannitol isotonic control group and NG group (*p* > 0.05).

### Effect of Yishen capsule serum on cell proliferation, and expression of beclin-1 and LC3-II in podocytes

**Figure 5.**
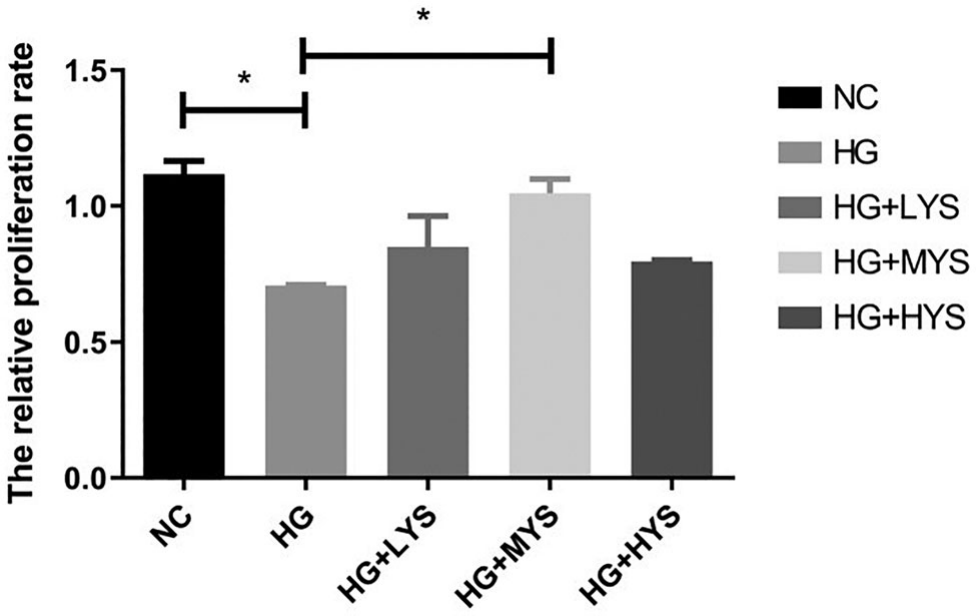
Effect of Yishen capsule serum on podocyte proliferation. Mouse podocytes were cultured under normal glucose (NG), high glucose (HG), high glucose + serum from low concentration of Yishen capsule (HG + LYS), high glucose + serum from medium concentration of Yishen capsule (HG + MYS) and high glucose + serum from high concentration of Yishen capsule (HG + HYS). Values are presented as the mean ± SD. *n* = 3. *p < 0.05.

As shown in Figure 5, proliferation of podocytes was inhibited by HG intervention (*p* < 0.05). Interestingly, Yishen capsule serum intervention reversed the inhibitory effect of HG on podocyte proliferation. Moreover, the maximum effect was observed when the serum was obtained from rats fed with medium concentration (1.25 g/kg/day) of Yishen capsule (*p* < 0.001). Therefore, serum from rats fed with medium concentration of Yishen capsule was used for further investigation and was named Yishen capsule serum.

**Figure 6.**
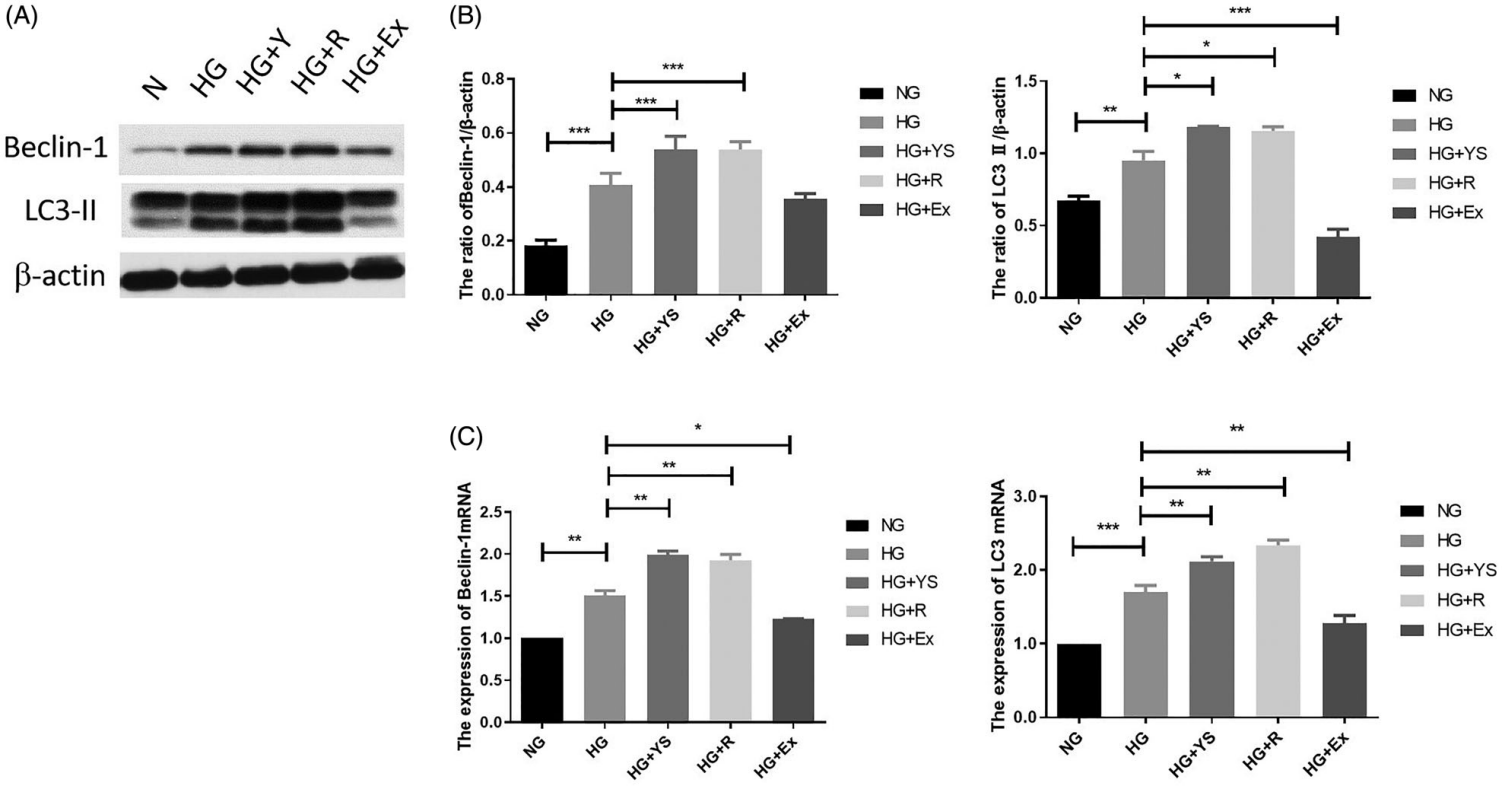
Effects of Yishen capsule serum on the expression of autophagy-related genes (i.e. Beclin-1 and LC3-II) under different interventions. NG = normal glucose, HG = high glucose, YS = Yishen capsule, R = resveratrol, and Ex = Ex-527. (A) Representative western blotting bands corresponding to Beclin-1 and LC3-II. (B) Quantification of Beclin-1 protein and mRNA. (C) Quantification of LC3-II protein and mRNA. Values are presented as the mean ± SD. *n* = 3. *p < 0.05; **p < 0.01; ***p < 0.001.

Compared to the control group, the expression levels of Beclin-1 and LC3-II were increased after HG stimulation (*p* < 0.001). Moreover, the expression of Beclin-1 and LC3-II in the Yishen capsule serum and resveratrol groups was higher than that in the HG group *(p* < 0.001), with no significant difference between the Yishen capsule serum and resveratrol groups (*p* > 0.05). However, the expression of Beclin-1 and LC3-II in the SIRT1 inhibitor (Ex-527) group was lower than that in the HG group *(p* < 0.05) (Figure 6).

### Effect of Yishen capsule serum on the expression of nephrin, SIRT1, and acetylated NF-κB p65 in podocytes

**Figure 7.**
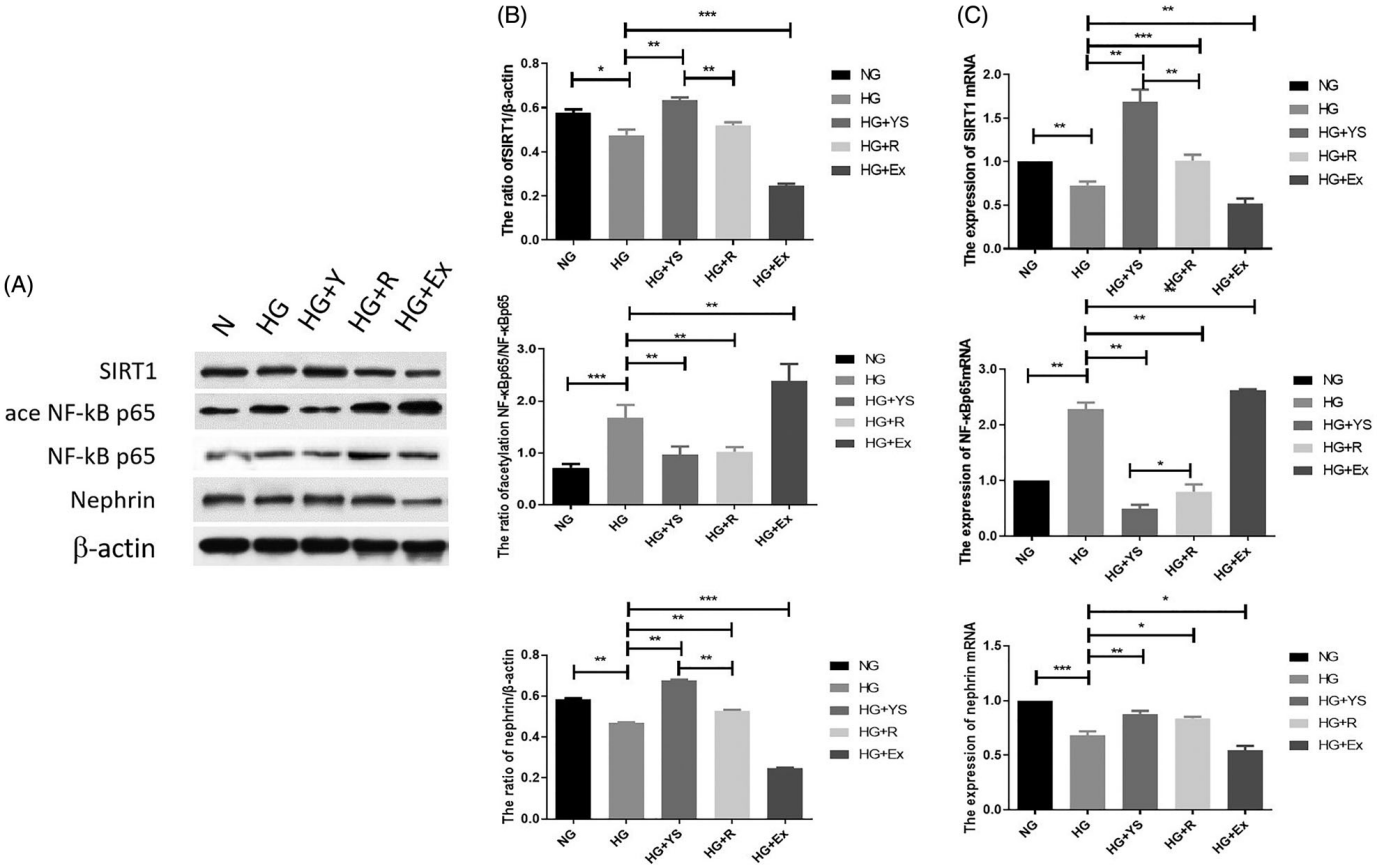
Expression of SIRT1, acetylated NF-κB p65, and nephrin under different culture conditions. NG = normal glucose, HG = high glucose, YS = Yishen capsule, R = resveratrol, and Ex = Ex-527. (A) Representative western blotting bands corresponding to SIRT1, acetylated NF-κB p65, and nephrin. (B) Quantification of protein levels of SIRT1, acetylated NF-kB p65, and nephrin. (C) Quantification of mRNA levels of genes. Values are presented as the mean ± SD. *n* = 3. *p < 0.05; **p < 0.01. ***p < 0.001.

**Figure 8.**
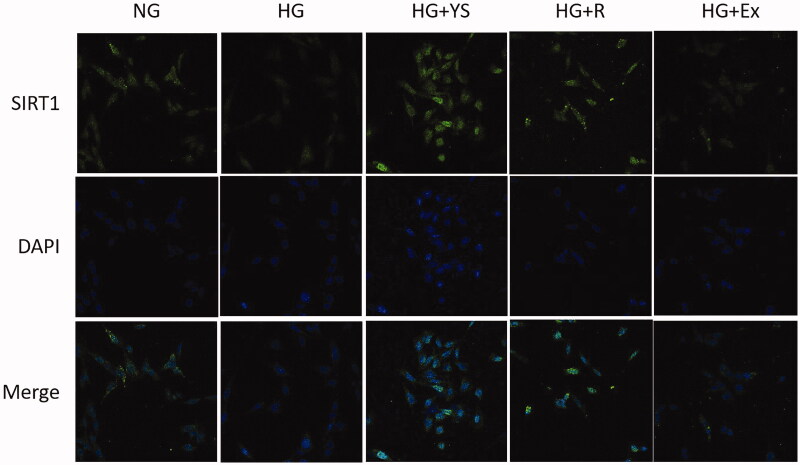
Representative immunofluorescence images showing enhanced nuclear translocation of SIRT1 in podocytes in the Yishen capsule serum and resveratrol groups and suppressed nuclear translocation of SIRT1 in the HG and SIRT1 inhibitor (Ex-527) groups. NG = normal glucose, HG = high glucose, YS = Yishen capsule, R = resveratrol, and Ex = Ex-527.

The mRNA and protein levels of SIRT1, acetylated NF-κB p65, and nephrin were further analyzed in the podocytes following different interventions. As shown in Figure 7, HG intervention for 24 h reduced both mRNA and protein levels of SIRT1 and nephrin. On the contrary, HG significantly induced mRNA expression of NF-κB p65 and enhanced the protein level of acetylated NF-κB p65. The expression of SIRT1 and nephrin in the Yishen capsule serum and resveratrol groups was higher than that in the HG group (*p* < 0.05), whereas the mRNA level of NF-κB p65 was lower than that in the HG group (*p* < 0.05). Rat serum containing Yishen capsule outperformed resveratrol in upregulating SIRT1 and nephrin expression, while downregulating acetylated NF-κB p65 expression in podocytes under HG conditions (Figure 7(C)). Moreover, the expression of SIRT1 and nephrin in SIRT1 inhibitor (Ex-527)*-*treated cells was lower than that in the HG group *(p* < 0.05), whereas acetylated NF-κB p65 and NF-κB mRNA levels were higher than that in the HG group (*p* < 0.05). To identify the underlying mechanism, the localization of SIRT1 in podocytes was examined. As shown in Figure 8, compared to the control group, Yishen capsule serum and resveratrol significantly facilitated the nuclear localization of SIRT1, while HG and SIRT1 inhibitor (Ex-527) suppressed the nuclear translocation of SIRT1 in podocytes.

### Electron microscopic observations

**Figure 9.**
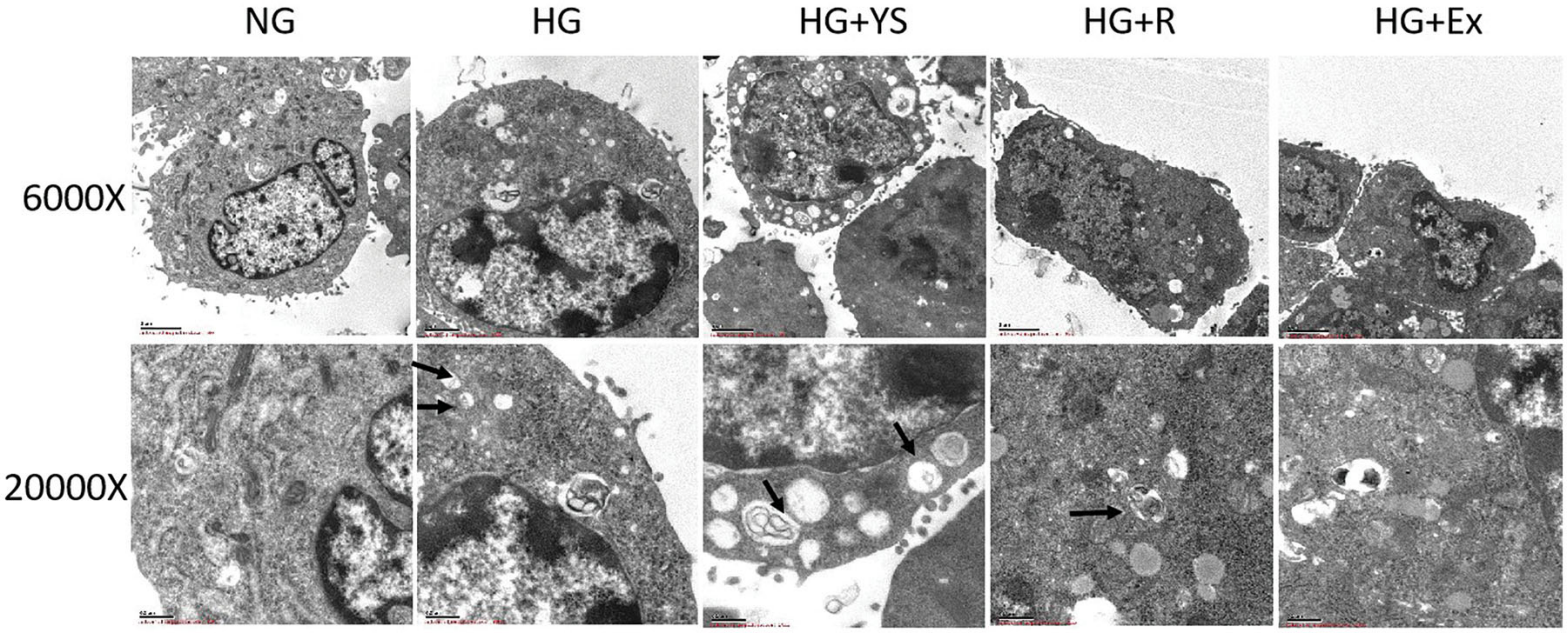
Representative transmission electron microscopic (TEM) images of podocytes under different culture conditions. NG = normal glucose, HG = high glucose, YS = Yishen capsule, R = resveratrol, and Ex = Ex-527. More autophagosomes are present in the HG treatment groups (i.e. HG, HG + Y, HG + R, and HG + E). Autophagosomes and vacuoles are increased in HG + Y and HG + R compared to that in the HG + E group. Images were captured at 6000 × (upper) and 20000 × (lower) magnification, respectively, with a transmission electron microscope. Black arrows indicate autophagosomes.

Microvilli-like structures on the cell surface and few autophagosomes in the cytoplasm were observed in the podocytes in the control group (Figure 9). HG treatment suppressed the formation of microvilli-like protuberances and induced the formation of autophagosomes in the cytoplasm of podocytes. Although Yishen capsule serum and resveratrol did not induce the formation of microvilli-like protuberance, autophagosome and vacuole formation in the cytoplasm were increased. In contrast, SIRT1 inhibitor (Ex-527) reduced autophagosomes, and induced the nucleus to become atypical.

## Discussion

DN is one of the most common complications of diabetes and a leading cause of end-stage chronic kidney disease. DN-associated morbidity is increasing annually. In the early stage, DN manifests clinically as microalbuminuria (defined as a urinary albumin excretion rate of 20–200 µg/min), followed by macroalbuminuria and renal failure with the progression of the disease. Symptomatic treatment including control of blood glucose, blood lipids, and blood pressure, is currently the main clinical strategy to delay the progression and related complications of the disease. Currently, the prevention and treatment of DN mainly focuses on the use of medications, such as angiotensin-converting enzyme inhibitors and angiotensin receptor blockers. However, their clinical efficacy in delaying DN progression is limited. Tremendous progress has been made in the treatment of DN using traditional Chinese herbal medicines, thus bringing hope to DN patients. Herein, we found that Yishen capsule that is mainly composed of five Chinese herbs (Astragalus membranaceus, Angelica sinensis, Euryale ferox, Alisma orientale, and Rhodiola rosea) improves DN by promoting autophagy through the regulation of the SIRT1/NF-κB pathway.

The progression of glomerulosclerosis in DN patients can be delayed by reducing urinary protein levels. Decreased urine protein amount, therefore, is accepted as an important indicator for the effective clinical treatment of DN. In this study, DN rats that were treated with Yishen capsule were found to have improved health condition, including decreased urine output and urine protein amount, attenuated glomerular hypertrophy, proliferation of mesangial cells, renal tubule dilation, and degeneration of renal tubule cells. In contrast, we observed a gradual aggravation of the condition of rats in the DN group, including loss of weight and hair, decreased mobility and responsiveness, increased diet and water intake, and increased urine output, similar to the clinical features of diabetic patients. These findings suggest that Yishen capsule improves DN. However, the blood glucose levels were not significantly downregulated in the rats treated with Yishen capsule and resveratrol, which may be because of the relatively short observation period.

Podocytes constitute one of the major cell types in the renal glomerulus. Under physiological conditions in the podocytes, a high degree of autophagic activity contributes to the degradation and clearance of damaged proteins as well as senescent organelles, thereby maintaining cell homeostasis [16,17]. Injury and dysfunction of podocytes play a very important role in DN progression, which makes them key therapeutic targets in DN patients [18]. Therefore, autophagic disorders, especially in podocytes, are considered to be associated with DN progression [2,3]. Alternations in autophagy, such as increased expression of autophagy-related proteins (i.e. LC3-II and Beclin-1) and increased formation of autophagosomes, have been observed in podocytes in STZ-induced DN rats and in mouse podocytes cultured in HG [19,20]. In this study, the expression of LC3-II and Beclin-1 was lower in the DN group than in the N group, suggesting the occurrence of autophagic disorder during DN progression. Increased formation of autophagosomes observed *in vitro* also confirmed autophagic disorder in this study. These findings are consistent with those reported by previous studies [21,22]. Resveratrol and genistein intervention increase the expression of LC3-II, reduce the expression of p62, activate autophagy, and promote podocyte repair *in vitro* [23,24]. Resveratrol is a natural SIRT1 activator. Current studies with resveratrol revealed its potential to improve DN *via* antioxidant effects or by activating SIRT1. Activated SIRT1 reportedly exerts protective effects through a variety of mechanisms, including autophagy [25–27]. In addition, Yishen capsule was found to ameliorate podocyte injury and delay the progression of DN by regulating the expression of nephrin, podocin, WT-1, α-actinin 4, and podocalyxin in the kidney and podocytes [10, 28,29]. In this study, Yishen capsule was found to show similar ability as resveratrol in enhancing LC3-II and Beclin 1 expression in the kidney. In a previous study, serum from rats treated with different concentrations of Yishen capsule was used to stimulate podocyte function *in vitro* [11]. Similarly, given that the actual effective components of Chinese medicines in the blood after metabolism *in vivo* are not completely the same as the components in the original herbs, we treated podocytes with serum containing Yishen capsule *in vitro*. The results from our *in vitro* experiments confirmed that Yishen capsule serum significantly induced podocyte proliferation, attenuated podocyte injury, increased the formation of autophagosomes, and enhanced the expression of LC3-II and Beclin-1. These results suggest that HG causes autophagy in podocytes, consistent with a previous report [19]. This phenomenon may result from a self-protective stress response, through which podocytes maintain their function and homeostasis by increasing autophagy within a short time after stimulation with HG.

SIRT1 [12] is a member of the Sirtuins (a gene family of the silent information regulator) family of proteins that are highly conserved during evolution. SIRT1 is involved in many physiological processes, such as metabolism, mitochondrial homeostasis, cell proliferation, autophagy, and apoptosis. Abnormal expression of SIRT1 has been found in podocyte injury during DN [30,31]. Moreover, podocyte injury and proteinuria were observed in a mouse model of podocyte-specific SIRT1 knockout [13]. In this study, the expression of SIRT1 was found to be enhanced in the kidneys of DN rats treated with Yishen capsule, suggesting a role of SIRT1 in the efficacy of Yishen capsule in DN. Treatment with a SIRT1 antagonist, Ex-527, decreased the expression of Beclin-1 and LC3-II in our *in vitro* experiments, in contrast to the effects of Yishen capsule serum. Expression of SIRT1 alleviated renal injury, and SIRT1 overexpression in degenerative nucleus pulposus cells regulated nuclear translocation of NF-κB, indicating a role of the SIRT1/NF-κB signaling pathway in renal injury [32]. In this study, reduced expression of acetylated NF-κB p65 was found in the kidneys of DN rats treated with Yishen capsule. Further *in vitro* investigation revealed that Yishen capsule serum reduced the expression of acetylated NF-κB p65. The regulation of NF-κB by Yishen capsule serum in the current study is consistent with the role of NF-κB in the pathophysiological process of DN [33,34]. However, in head and neck squamous cell carcinoma Ca27 cells, activation of NF-κB p65 upregulated the expression of LC3-II and Beclin-1 [15]. Moreover, autophagic function was attenuated by inhibiting IκB degradation and reducing NF-κB p65 phosphorylation [35]. Furthermore, blocking NF-κB activation in podocytes cultured in HG medium hindered the upregulation of the podocyte autophagy protein, LC3-II [36]. The current study demonstrated that increased expression of SIRT1 and decreased expression of acetylated NF-κB by Yishen capsule serum significantly enhanced autophagy function. We also found that the regulation of autophagy by Yishen capsule serum was dependent on its ability to regulate SIRT1 and acetylate NF-kB p65, indicating a role for the SIRT1/NF-κB signaling pathway in regulating autophagy. Similarly, Yishen capsule was found to improve podocyte injury in rats with DN by decreasing urine protein, increasing SIRT1, LC3-II, and Beclin-1 expression both *in vitro* and *in vivo*. In contrast, rat serum containing Yishen capsule outperformed resveratrol in upregulating SIRT1 and nephrin expression, while downregulating acetylated NF-κB p65 expression in podocytes under HG conditions *in vitro*.

Drug concentrations and pharmacokinetics were not explored in this work. Because the complex composition of Yishen capsule indicates that the complex active components after metabolism and difficult detection in the body. In conclusion, the present study demonstrates that Yishen capsule improves DN by promoting autophagy through regulating the SIRT1/NF-κB pathway. The underlying mechanisms identified in this study will enhance the clinical application of Yishen capsules and provide guidelines for developing new therapeutic agents for DN patients.

## Data Availability

The datasets generated and/or analyzed during the current study are not publicly available due to data sharing agreements but are available from the corresponding author on reasonable request.

## Abbreviations

ACEI: angiotensin-converting enzyme inhibitor
ARB: angiotensin receptor blocker
CCK-8: cell counting kit-8
DAPI: 4,6-diamino-2-phenyl indole
DMEM: Dulbecco's modified eagle medium
DN: diabetic nephropathy
FITC: fluoresceine isothiocyanate
JAK/STAT: Janus kinase/signal transducers and activators of transcription
LC3: microtubule-associated protein 1 light chain 3
MPC: mouse podocyte
NF-κB: nuclear factor-kappa B
qPCR: quantitative real-time polymerase chain reaction
SIRT1: silent mating type information regulation 2 homolog-1
SPF: specific pathogen free
STZ: streptozotocin
WT-1: The Wilms' tumor gene product
